# Characterization of Human Recombinant β1,4-GalNAc-Transferase B4GALNT1 and Inhibition by Selected Compounds

**DOI:** 10.3390/molecules30173615

**Published:** 2025-09-04

**Authors:** Iram Abidi, Alexander N. Kocev, Jonathan L. Babulic, Chantelle J. Capicciotti, Jagdeep Walia, Inka Brockhausen

**Affiliations:** 1 Department of Biomedical and Molecular Sciences, Queen’s University, Kingston, ON K7L 3N6, Canada; 2 Department of Chemistry, Queen’s University, Kingston, ON K7L 3N6, Canada; 3 Department of Pathology, Queen’s University, Kingston, ON K7L 3N6, Canada

**Keywords:** B4GALNT1, GalNAc-transferase, GM2 gangliosidosis, inhibition, bioinformatics

## Abstract

Gangliosides are essential for membrane functions, cell recognition, and maintenance of the nervous system. GM2 gangliosidosis is a group of rare genetic lysosomal storage diseases that includes Tay-Sachs disease (TSD), Sandhoff disease (SD), and AB variant. TSD and SD are characterized by deficient β-N-acetyl-hexosaminidase activity. This leads to decreased catabolism of β-N-acetyl-hexosamine-containing ganglioside GM2 in the lysosomes, damage to cells and tissues, and severe neurological symptoms. GM2 is a major ganglioside accumulating in TSD and SD, and is synthesized from GM3 by β1,4-N-acetylgalactosaminyltransferase 1 (B4GALNT1, GM2 synthase). Therapies under development for GM2 gangliosidosis include adeno-associated virus gene therapy, enzyme replacement, and substrate reduction therapy (SRT). The goal of this work was to express and purify human B4GALNT1, characterize its activity, and explore its structural features by protein modeling and substrate docking. We used a panel of synthetic compounds to study their potential inhibition of B4GALNT1 activity. This work can serve to develop SRT for GM2 gangliosidosis.

## 1. Introduction

**Figure 1:**
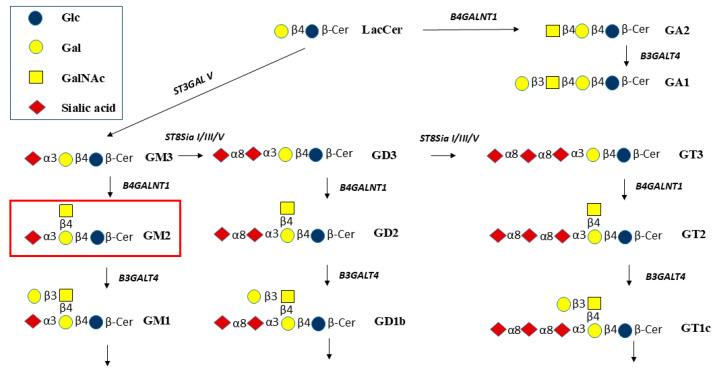
Biosynthetic pathways from lactosylceramide (Lac-Cer) to GA2 and GA1 and to gangliosides GM3, GM2, and GM1, and to gangliosides having more than 1 sialic acid residue. B4GALNT1 is required to synthesize GA2, GM2, GD2, and GT2. The GalNAc residue is then extended by Galβ1-3. Galβ1-4 and Galβ1-3 residues may be substituted by sialic acid residues (modified from [10]).

Glycosphingolipids (GSLs) are integral components of the cell membrane and are especially rich in the brain as sialic acid (Sia)-containing gangliosides [1] that are based on a lactosyl-ceramide (Lac-Cer, Galβ1-4Glcβ-Cer) core (Figure 1). GSLs are concentrated in membrane microdomains (lipid rafts). They help to maintain membrane fluidity and integrity, they act as cell surface epitopes and markers, participate in the functions and folding of membrane proteins and receptors, and help in cell–cell recognition and communication and regulation of the immune system. Gangliosides play crucial roles in early childhood and brain development and are involved in the maintenance of the nervous system [2,3,4,5].

In addition, many GSLs are receptors for bacterial toxins. For example, GM2 (GalNAcβ1-4Galβ1-4Glcβ-Cer) was shown to bind to Delta-toxin from *Clostridium perfringens* [6]. The simplest ganglioside is GM3, Siaα2-3Galβ1-4Glcβ-Cer, which is converted in the Golgi by an α2,8-sialyltransferase (e.g., ST8SiaI) to GD3, Siaα2-8Siaα2-3Galβ1-4Glcβ-Cer. GM3 and GD3 are prominent gangliosides found in the central nervous system. GD3 is upregulated in many invasive ductal breast carcinoma cases [7]. GD3 is also the predominant ganglioside in neural stem cells and is important for neuronal functions [8]. GD3 associates with epidermal growth factor receptor in breast cancer cells, and both GD3 and GD2 (Siaα2-8Siaα2-3[GalNAcβ1-4] Galβ1-4Glcβ-Cer) were found to be involved in signal transduction [7]. GD2 is also considered a marker for mesenchymal stromal cells [9].

GM3 and GD3 are converted by β1,4-GalNAc-transferase B4GALNT1 to form GM2 and GD2, respectively (Figure 1). Thus, B4GALNT1 has been named GM2 synthase or GD2 synthase. The enzyme also transfers GalNAc to Lac-Cer to form GA2, GalNAcβ1-4Galβ1-4Glcβ-Cer, and is expected to synthesize GT2 from GT3.

In the lysosomal catabolic pathways, the GalNAc residues are cleaved by β-*N*-acetyl-hexosaminidase (HEX) (Figure 1) [10]. Substrates for HEX include GA2, GM2, GD2, and GT2. Lacto- and neolacto-series and Gal-Cer series of GSLs also include structures having terminal GlcNAcβ1-3 residues, while Gb4 of the globo-series has a terminal GalNAcβ1-3. These GSLs could also be cleaved by HEX [11]. However, in lysosomal storage diseases (LSD) such as GM2 gangliosidosis, Tay-Sachs disease (TSD), and Sandhoff disease (SD), the cleavage of GalNAc is decreased or blocked due to mutations of HEX or an essential activator protein, GM2A. As a consequence, primarily GM2 accumulates in the brain of children, leading to progressive neurodegeneration and early death. Lyso-GM2 is the product of *N*-deacylase that cleaves the fatty acid residue of GM2 and other GSLs. Thus, with a high concentration of GM2, lyso-GM2 also accumulates. GD2 positivity in bone marrow cells may represent a useful prognostic marker for patients with non-metastatic neuroblastoma [12], and B4GALNT1 has been suggested to be a pan-cancer biomarker [13]. An inhibition of GM2 synthesis may thus be useful to study the role of GM2/GD2 in human tumor cells (malignant melanoma lines, neuroblastoma lines, and some glioma lines) that express high levels of B4GALNT1 mRNA [14]. Blocking the pathway to GM2 synthesis causes a deficiency of the next metabolites in the pathway, such as GM1, which has been shown to be linked to Parkinsonian symptoms in *B4galnt1* knock-out mice. The GM1 oligosaccharide without the Cer moiety was used to successfully treat these symptoms [15]. Thus, the functions of membrane-bound gangliosides are based on both the glycan and lipid moieties and their interactions with proteins.

Therapy for GM2 gangliosidosis is currently being developed by introducing the correct HEXA and/or HEXB coding genes through adeno-associated virus (AAV)-based gene therapies [16,17]. In a mouse model of HEX-deficiency, a dose-dependent correction of GM2 accumulation in the brain was achieved after AAV9 transfer. In addition, mice lacking GM2A also responded to AAV-based gene transfer [18]. An alternative potential treatment for GSL accumulation is enzyme replacement. For example, in Gaucher disease (glucoceramidase deficiency), a functional, modified enzyme can be repeatedly injected into patients to minimize the effect of GSL accumulation. However, these enzymes do not cross the blood/brain barrier, and there may be side effects; the underlying, progressing pathology is treated peripherally but not in the brain, and it is not a cure. The delivery of Golgi- and lysosomal-resident enzymes is difficult, since proper post-translational modifications to avoid immunogenicity and protection from protease digestion, as well as for targeting, may be required.

Another possibility for LSD treatment is substrate reduction therapy (SRT), which can reduce biosynthetic precursors and can, thus, reduce pathogenicity due to accumulation of GSL [19,20]. The Glc derivative *N*-butyldeoxynojirimycin (Miglustat) [21] functions as a competitive and reversible inhibitor of the enzyme glucosylceramide synthase (GCS) that forms Glc-Cer and is used to reduce the synthesis of GSL and ganglioside metabolites based on Glc-Cer. Miglustat is a treatment for Gaucher disease, but also leads to serious side effects. Another potential treatment to correct genetic abnormalities is based on cell therapy with induced pluripotent stem cells (iPSCs). iPSCs offer disease models and can also be developed into established healthy cell populations to be administered to patients. Although there is promise in these therapies, currently, there is no established cure for TSD and SD.

B4GALNT1 is a type-II Golgi transmembrane homodimeric protein [22] and belongs to the CAZy GT12 family of glycosyltransferases (GTs), having a GT-A fold and an inverting mechanism [23]. In humans, B4GALNT1 mutations that lead to decreased enzyme activity are associated with autosomal recessive spastic paraplegia, termed SPG26, a slowly progressive neurodegenerative disorder with increasing muscle weakness (Table 1) [24,25,26].

In mice lacking B4GALNT1 and GM2, the symptoms include Parkinsonian neuropathology [27], suggesting that B4GALNT1 is an essential enzyme. Forced expression of B4GALNT1 in human melanoma cells increased GM2 levels as well as cleavage of amyloid precursor protein that is associated with the pathophysiology of Alzheimer’s disease (AD) [28]. Levels of GM3 are increased in the brains of AD patients and in a mouse model [29]. Kaya et al. [5] determined by mass spectrometry that gangliosides GM3, GM2, and GM1 accumulate in amyloid plaques of AD mice. A GCS inhibitor that penetrates into the brain was shown to reduce GM3 levels as well as amyloid plaques in AD mice [30]. Inhibition of GlcCer synthesis would also reduce the levels of GM2 and other metabolites, and this could be beneficial in dementia. Thus, balanced levels of gangliosides play a critical role in cell membrane functions, and a complete inhibition of B4GALNT1 may lead to neuropathology. SRT should, therefore, aim to maintain an optimal level of enzyme activity and maintain a balance of gangliosides.

The primary objective of this study is to express, purify, and characterize recombinant human B4GALNT1 and to advance the development of appropriate inhibitors that reduce GM2 synthesis and thus may prevent pathological accumulation of GM2.

## 2. Results and Discussion

### 2.1. Protein Expression and Purification

The expression of recombinant His_8_-tagged B4GALNT1 in HEK293 and HEK293T cells showed very low levels and low enzyme activity. SDS-Page and Western blots indicated that the enzyme was associated with the pellet in an insoluble form and showed 0.01 nmol/h/mg with GM3 acceptor in the lysed pellet. We, therefore, used Expi293 suspension cells that were expected to produce large amounts of soluble recombinant protein. Using the Expi293 cell expression system, Western blot of the medium revealed a protein band at 88 kDa on Day 2, which became strongest by Day 5 of transfection. The lysed cell pellet showed no protein band at 88.8 kDa (Figure S1), indicating that soluble His_8_-B4GALNT1 was secreted into the culture medium with a maximal production on Day 5.

Purification of His_8_-B4GALNT1 was performed using Ni-NTA chromatography. Most of the enzyme protein eluted with 500 mM imidazole (Figure S2). B4GALNT1 appeared as a strong band on a 12% SDS-PAGE gel in elution fractions, corresponding to the theoretical size of 88.8 kDa, with a protein concentration of 0.4 mg/mL. Purified enzyme was concentrated using a 10MWCO Centricon column.

### 2.2. Characterization of Purified B4GALNT1 Activity

**Figure 2:**
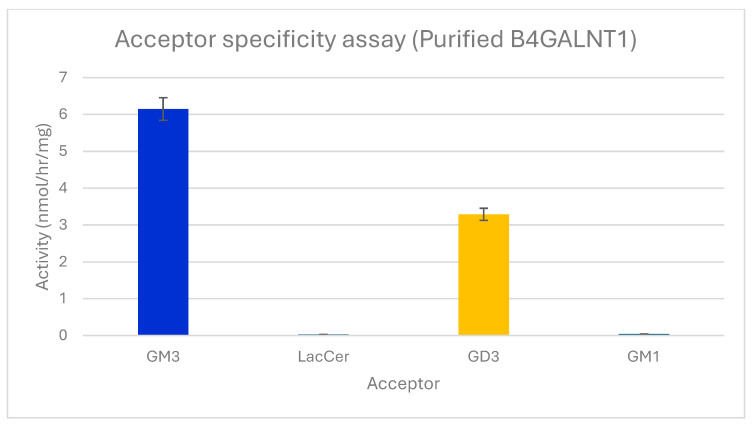
Acceptor substrate specificity of purified B4GALNT1. The activities of the enzyme are shown using 0.2 mM acceptor substrates GM3, Lac-Cer, GD3, or GM1 in standard assays. Error bars show variation between duplicate assays. All values were standardized against the negative control that lacks acceptors. Only GSLs having a Siaα2-3Galβ1-4 sequence served as significant acceptors.

The activity of B4GALNT1 produced in Expi293 cells was confirmed with 0.2 mM GSL acceptor substrates using crude enzyme from the medium or highly purified enzyme. GM3 produced 6.1 nmol/h/mg activity with purified enzyme, while GD3 produced 3.2 nmol/h/mg. Lac-Cer and GM1, Galβ1-3GalNAc1-4(Siaα2-3)Galβ1-4Glcβ-Cer (Figure 1), showed <0.1 nmol/h/mg activity (Figure 2). This indicates that the Siaα2-3Galβ1-4 linkage in the acceptor is important for enzyme recognition, but an additional Sia residue in GD3 is less preferred. Thus, B4GALNT1 activity with GT3 acceptor is expected to be very low, although this remains to be tested in vitro. The activity was maximal with purified enzyme using GM3 as its major acceptor substrate. The apparent K_M_ for GM3 substrate with purified enzyme was 0.5 mM. This value is within the range reported for other B4GALNT1 activities from membrane fractions. The apparent K_M_ values reported for GM3 acceptor varied from 0.69 mM in bovine thyroid extracts [31] to 0.1 mM [32] for the rat enzyme and 0.5 mM for the human enzyme [33]. The absolute activities and K_M_ values, however, depend on the type of enzyme (membrane-bound or purified, type of construct), the expression system, and the conditions of the assay, including length of incubation and the type and concentration of detergent and cofactors. Thus, it has been difficult to determine an accurate apparent V_max_ value. In a cell extract, different species of the same enzyme and potentially different variants may be present.

The human enzyme can be proteolytically released as a soluble form [22]. Previous studies with various forms of B4GALNT1 from human, mouse, and rat sources also showed that GM3 is the major acceptor substrate and GM2 is, therefore, the major GSL product of B4GALNT1 [4,22,31,32,33,34,35,36]. The rat enzyme from ascites hepatoma cells, for example, has 77.3% activity towards GD3 compared to GM3 and only 1.2% activity with LacCer [34]. A high B4GALNT1 activity was achieved by Welland et al. [37] using a liposome-based assay with the enzyme tethered to the membrane via a His-tag.

Several nucleotide sugars were tested as donor substrates for B4GALNT1 with GM3 acceptor, replacing UDP-GalNAc in standard assays. UDP-GalNAc was the only active donor substrate while UDP-Gal, UDP-Glc, and UDP-GlcNAc failed to serve as donor substrates. Thus, B4GALNT1 has a strict donor specificity.

Although recombinant B4GALNT1 appeared to be soluble when expressed in Expi293 cells, Kyte and Doolittle hydrophobicity analysis revealed several shorter hydrophobic sequences that may possibly be involved in binding the hydrophobic moiety of the acceptor substrates. These hydrophobic sequences may also be responsible for an accumulation of misfolded protein in the pellet when expressed in HEK cells. We, therefore, tested whether detergent that may aid the solubility of the substrate or affect the folding of purified B4GALNT1 could improve the activity. The activity increased slightly from 0.06 to 0.12% Triton X-100 in the assay (Figure S3). Although the enzyme has previously been assayed with various types of detergents up to 2% [22], we used 0.06% Triton X-100 in order to prevent problems with GSL product isolation after the incubation. CDP-choline was previously used in B4GALNT1 assays as a potential inhibitor of UDP-GalNAc degradation [32]. In our standard assays of highly purified B4GALNT1 that should lack hydrolytic enzymes, the presence of 10 or 30 mM CDP-choline did not inhibit or activate B4GALNT1 activity and was therefore omitted in further assays.

**Figure 3:**
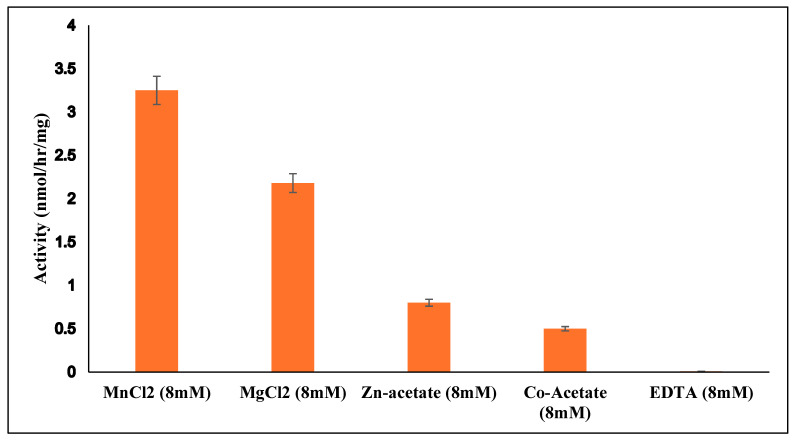
Dependence of purified B4GALNT1 activity on divalent metal ions. Mn^2+^ ions in the standard assay with 0.2 mM GM3 acceptor were replaced by other divalent metal ions or EDTA. All values were standardized against the negative control. Error bars indicate the variation between duplicate assays.

Generally, buffer pH values have been used in B4GALNT1 assays between 6.6 and 7.6 [22,32,33] while we used pH 7 due to a broad pH optimum in our assays. B4GALNT1, as an inverting GT12 enzyme, may require the presence of metal ions for donor binding and catalysis. The effect of metal ions was tested with 8 mM MnCl_2,_ MgCl_2,_ Zn-acetate, Co-acetate, or EDTA in standard assays of B4GALNT1 activity using GM3 as acceptor substrate. Compared to MnCl_2_, MgCl_2,_ Zn-acetate and Co-acetate resulted in activities of 67%, 22%, and 15% respectively, while assays conducted with EDTA resulted in complete loss of activity (Figure 3). This showed the dependency of B4GALNT1 activity on divalent metal ions, with Mn^2+^ being optimal. In contrast, the membrane-bound rat B4GALNT1, which has about 97% sequence identity to the human enzyme, has been reported to have higher activity in the presence of 10 mM Co^2+^ compared to Mn^2+^ [32].

### 2.3. Bioinformatics

The CAZy data bank classified B4GALNT1 into the GT12 family, characterized as inverting GTs having a GT-A fold and one Rossmann domain where the central beta-sheet is expected to contain the active site. Only four GT12 enzymes have been characterized, and these are all GalNAc-transferases from man, mouse, and rat with distant similarity to GT2, GT21, and GT27 families. The two subunits of B4GALNT1 homodimers have two catalytic domains in antiparallel orientation [36]. B4GALNT1 is disulfide bonded with C429-C476, forming an intra-chain bond, and C80-412 and C82-C529 forming inter-subunit bonds [36,37].

**Figure 4:**
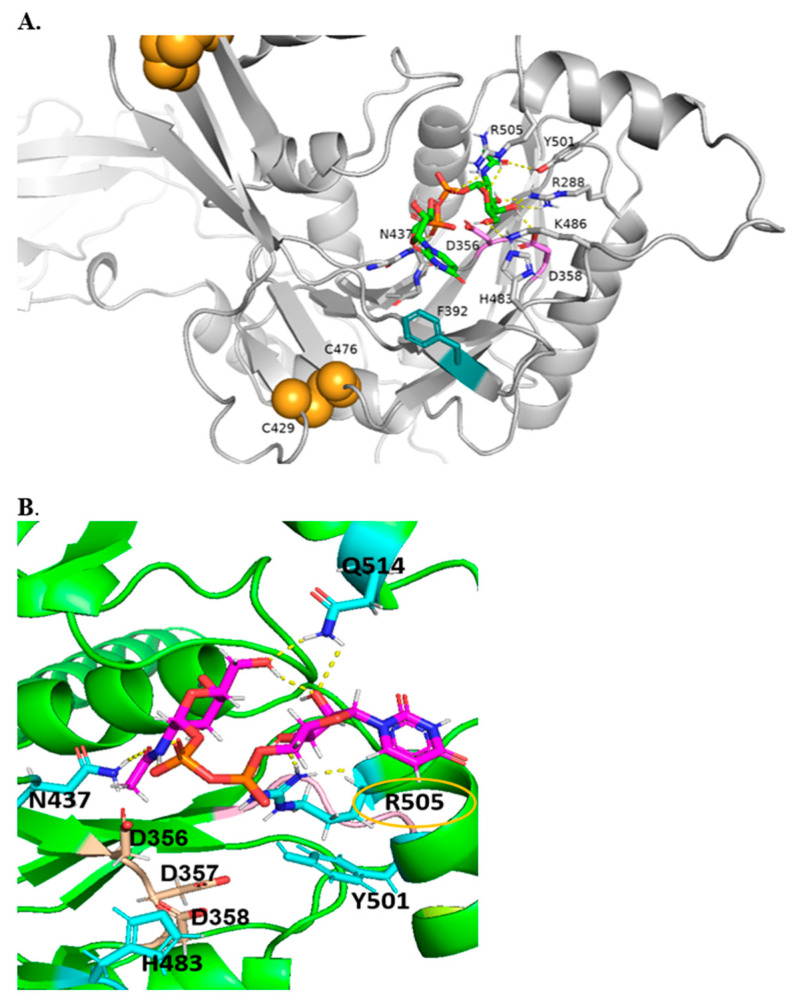
Alphafold2 models of B4GALNT1 with DXD motif in magenta and intrachain disulfide bonds in orange. (**A**). Global view of multi-domain B4GALNT1. (**B**). Model of B4GALNT1 docked with donor UDP-GalNAc making predicted interactions with ligand shown with labels, with yellow dashed lines representing H-bonds and potential pi–pi stacking partner shown in teal.

All of these Cys residues are highly conserved, suggesting that dimer formation and three-dimensional protein structure are essential for activity (Figure 4). Human B4GALNT1 has a high degree of amino acid sequence identity with the enzyme from mouse (86.87%), rat (87.62%), bovine (90.99%), and Macaca fascicularis (98.87%). All of the Cys residues involved in disulfide bonding, as well as a putative catalytic DDD sequence, are present in these species.

**Figure 5:**
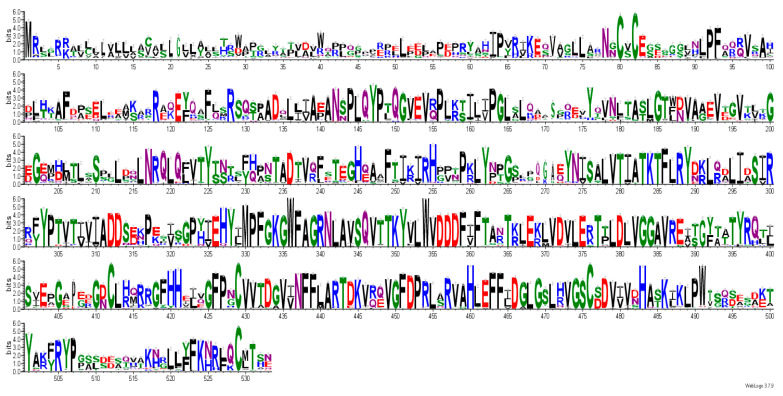
Consensus sequence logos generated by Weblogo. Sequence conservation in full-length B4GALNT1, showing conserved residues as large letters. Among highly conserved residues are D356 (DxD motif), Cys80, Cys82, Cys412,Cys 429, Cys476, Cys529 (disulfide bonds), R300 and F439 (SPG), and N179, N274 (N-glycosylation sites).

Table 1 lists the conserved amino acids that may represent important residues for the biological function of B4GALNT1. The consensus sequence aligned by MUSCLE using 352 aligned protein sequences and generated with WebLogo is shown in Figure 5. Two centrally located DxD sequences are found in GM2 synthases. However, only D356, D357, and D358 are highly conserved sequences in GM2 synthases that line the active site, and D356 may be the critical Asp residue involved in catalysis [23]. DxD is a widely conserved motif in GT-A folded enzymes and is thought to coordinate with metal ions and play a role as a catalyst (Table 1) [38]. Modeling showed that the DxD motif (D356 to D358) is within the catalytic site, explaining that mutations of D356 and D358 abolished activity. The DD sequence is also highly conserved in B4GALNT1. Inamori et al. [39] identified an inactive SPG variant of D313, the second Asp of the DD sequence, that is thought to bind UDP-GalNAc. Other inactive SPG variants of highly conserved residues include N437K, A441E, P453H, and R519W. However, the F438L mutant exhibited low (<10%) activity.

Interestingly, the V352A mutant of B4GALNT1 had 167% activity compared to the wild type and may facilitate D356-mediated catalysis, possibly by widening the substrate binding groove. In contrast, the W354A mutant was only 23.5% active [38].

Other conserved amino acids that appear to be within the active site are highly conserved N437, H483, Y501, and R505. Welland et al. [37] suggested that R505 is coordinated with UDP in acceptor and product, likely by binding to phosphate groups. Several variants of B4GALNT1 were found in SPG patients (Table 1). Conserved variant amino acids include R300, D433, and F439, but according to modeling, are not located within the active site. Less conserved variant amino acids include A516, R519 in SPG, and the roles of these residues are unknown. Interestingly, mutations in SPG26 [24] have been identified for residues R300 (to C) and D433 (to A). Since these may not be critical for catalysis or UDP-GalNAc binding, they may be involved in shaping the overall substrate binding site for UDP-GalNAc.

The amino acids near the proposed substrate binding and catalytic site in our study align well with those suggested by Welland et al. [37] based on the crystal structure and molecular dynamics simulations of the dimer of the soluble lumenal domain of B4GALNT1. The proposed catalytic domain encompasses amino acids 258 to 533. Even in the absence of the tm domain, amino acids flanking the active site were suggested to form surface loops that contribute to membrane insertion.

There are three potential N-Glycosylation sites at N79, N179, and N274 that are distant from the UDP-GalNAc binding site. Mutations in these amino acids led to decreased activity [40]. Only N179 and 274, but not N79, are highly conserved and may contribute to maintenance of protein structure. Some of these N-glycans are sialylated and could contribute to the overall folding, dimer formation, and stability of the enzyme in the late Golgi compartment [22].

It is not yet known if any of the Thr and Ser residues of B4GALNT1 carry O-glycans. For example, S314 and S401 are potential O-glycosylation sites [41]. HEK cells were previously shown to have the enzymes that form sialylated core 1 and 2 O-glycans as well as complex N-glycans [42] and could modify B4GALNT1.

### 2.4. Molecular Docking

Figure 4 shows a model of B4GALNT1 docked with the essential donor substrate UDP-GalNAc and suggests that conserved N437, R505, R288, and K486 are engaged in hydrogen bonding interactions with donor UDP-GalNAc and could be associated with the UDP reaction product. In addition, H483 and Y501 are near the UDP-GalNAc binding site and may be crucial residues for other interactions. The proximity of the F392 side chain to the uridine moiety suggests the possibility of coordination via pi–pi stacking. The DxD motif (356-358) that can coordinate Mn^2+^ ion is in proximity to Y501, which may be necessary for coordinating the hydrophobic acceptor substrate. R505 is coordinated near C1 of the GalNAc moiety and is mutated in GM2 synthase deficiency [25] (Figure 4). It seems likely that R505 and H483 are also involved in binding sialylated acceptors such as GM3. As with other GTs, it was not possible to successfully dock the acceptor substrates into the protein, likely because of the complexity in binding a ceramide moiety, as well as a complex glycan chain.

### 2.5. Inhibitors

**Figure 6:**
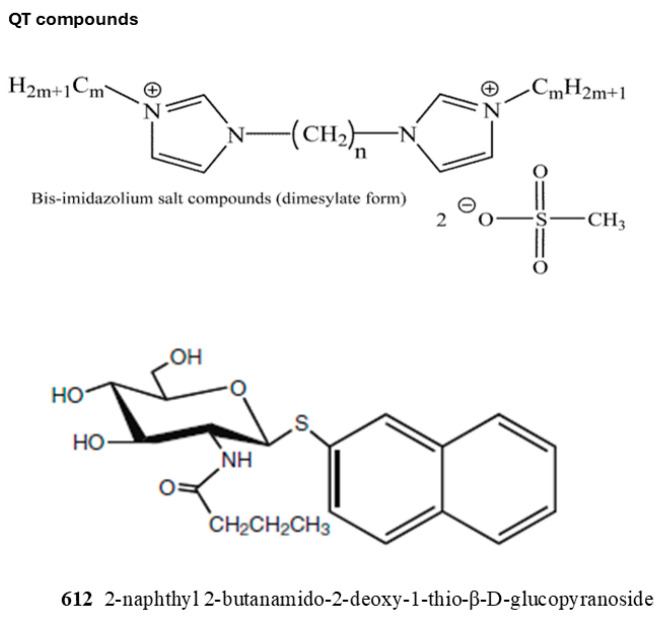
The structures of inhibitors used in this study are shown. The effects on activity are listed in Table 2. For QT compounds: **n**, number of aliphatic core carbons; **m**, number of symmetrical aliphatic carbons attached to the imidazolium rings.

Currently, inhibitors for B4GALNT1 are not available. We tested several synthetic compounds as inhibitors of B4GALNT1 activity, but many of them did not inhibit the activity. These compounds are not directly related to structures of B4GALNT1 substrates but could potentially bind to the protein via hydrophobic or electrostatic interactions. A series of compounds named QT [43] showed various degrees of inhibition. QT compounds have two positively charged bis-imidazolium rings connected to an aliphatic spacer between rings (**n**) and symmetrical aliphatic wing chains (**m**) attached to the nitrogen of imidazolium groups. The highly flexible aliphatic chains have varying numbers of carbons, and the compounds are in dimesylate salt forms (Table 2, Figure 6). QT compounds with a higher number of carbons in the aliphatic chains previously showed inhibition for selected enzymes, and this was unrelated to the type of substrates used [43]. Another potential inhibitor was 2-naphthyl 2-butanamido-2-deoxy-1-thio-β-D-glucopyranoside (**612**). GlcNAc-naphthyl derivatives such as **612** were shown to inhibit bovine milk β1,4-Gal-transferase as acceptor substrate analogs [44,45].

To determine inhibition, purified B4GALNT1 was assayed under standard assay conditions utilizing 0.2 mM GM3 as the acceptor substrate, 0.39 mM UDP-GalNAc as donor substrate, and 1 mM **QT** compound or **612**. Table 2 summarizes the variable inhibitory effects of **QT** compounds on B4GALNT1 activity. Compound **612** showed 44% inhibition. **QT163** (6/11), having a 6-carbon spacer chain and 11-carbon wing chains, showed 80% inhibition, with an IC_50_ value of 0.2 mM. Other **QT** compounds inhibited the activity between 40 and 94%. Our results suggest that it is not the length of either the core or the wing aliphatic chain that determines the inhibitory potential, but a combination of both and, likely, the overall properties of the compounds. In future studies, we will try to dock **QT163** to the B4GALNT1 model to determine which hydrophobic or negatively charged groups in the enzyme could be responsible for inhibitor binding. This binding is expected to affect the structure of the catalytic domain or block the access of substrates.

B4GALNT1 assays were carried out with 0.2 mM GM3 as the acceptor, 0.07 mM donor, and 1 mM inhibitor concentration. The values shown in the Table are averages of at least duplicate determinations. The structures of inhibitors are shown in Figure 6. Number of aliphatic core carbons (**n**); number of symmetrical aliphatic carbons attached to the imidazolium rings (**m**).

## 3. Materials and Methods

### 3.1. Materials

Materials were purchased from Sigma-Aldrich (Burlington, MA, USA), unless otherwise stated. UDP-[^3^H]GalNAc, UDP-[^3^H]Gal, UDP-[^3^H]GlcNAc, and UDP-[^14^C]Glc were obtained from American Radiolabeled Chemicals (St. Louis, MO, USA). GSLs were from Cayman: Ann Arbor, MI, USA. Potential inhibitors were synthesized as described [46]. FBS was from Wisent: Saint-Jean-Baptiste, QC, Canada.

### 3.2. Plasmid Isolation and Transformation

The plasmid pGEn2-DEST was developed and donated by Kelly Moremen, University of Georgia. This mammalian expression vector has a CMV promoter, N-terminal His_8_ tag, AviTag, Super GFP tags, and ampicillin resistance genes. Sequences were confirmed by Sanger sequencing (The Centre for Applied Genomics, Toronto, ON, Canada). To enhance protein solubility, the short cytoplasmic and the transmembrane (tm) domain (amino acids 1–25) at the N-terminus were deleted in the recombinant B4GALNT1 construct.

Plasmid pGEn2-DEST was transformed into *E. coli* (DH5α) using the heat shock method. Positive transformants were selected from LB (Lennox)-agar plates containing 100 µg/mL ampicillin (pGEn2-DEST). An overnight small-scale culture from a single colony was prepared in 3 mL of LB Broth (BioShop) with 100 µg/mL ampicillin, maintained at 37 °C, 225 RPM for 16 h. An aliquot of the overnight culture (100 µL) was added to a 250 mL Erlenmeyer flask, containing 100 mL of LB broth and 100 µg/mL ampicillin. The flask was shaken at 37 °C, 225 RPM, to reach absorbance OD600 = 0.6.

For isolation of high-quality plasmid DNA from *E. coli* DH5α cultures, Gene JET Plasmid Midiprep Kit (Thermo Scientific, Waltham, MA USA) was used. The above prepared 100 mL culture was centrifuged for 10 min, and supernatant was discarded. The bacterial pellet was resuspended using 2 mL Resuspension Buffer, and the suspension was vortexed. The plasmid quality and quantity of the isolated DNA were examined using a NanoDrop spectrophotometer. The total yield was 145 µg DNA per 100 mL culture. Plasmid DNA was filtered through a 0.22 µm filter prior to transfection.

### 3.3. Expression of B4GALNT1 in Expi293 Cells

The mammalian cell-based expression system of human embryonic kidney (HEK) Expi293 suspension cell lines was used to express soluble protein in sufficient quantities for characterization, based on previously described protocols [47]. Expi293 cells were cultured in expression medium (Thermo-Fisher, Waltham, MA, USA). Cells were maintained in a humid 5% CO_2_ atmosphere at 37 °C, shaking at 120 RPM. Cells were passaged for maintenance after reaching 4 × 10^6^ viable cells/mL. Cells were cultured for at least 3 passages following thaw prior to transfection. Expi293 cells were transiently transfected with the pGEn2-DEST plasmid containing the human B4GALNT1 gene [48] using Expifectamine^TM^ 293 Transfection Kit (Thermo-Fisher, Waltham, MA, USA).

On the day of transfection, the cells were diluted to 3 million cells/mL in 100 mL of culture medium. Plasmid DNA containing the B4GALNT1 gene (100 µg) was diluted with 5.6 mL of Opti-MEM GIBCO medium (Thermo-Fisher, Waltham, MA, USA). A total of 320 µL of Expifectamine lipid-based transfection reagent was diluted with 6 mL of Opti-MEM GIBCO medium. The diluted transfection agent was added to the DNA dropwise and mixed gently by inverting the tube 4 times, followed by incubation for 15 min at room temperature. The pre-complexed DNA and Expifectamine mixture was then added dropwise to the cells, swirling gently. Transfected cells were then maintained in a humid 5% CO2 atmosphere at 37 °C, shaking at 120 RPM. Expifectamine^TM^ 293 Transfection Kit (Thermo-Fisher, Waltham, MA, USA) enhancer reagents were added 16 h following transfection. Cell aliquots were taken daily to monitor cell growth and viability with a hemocytometer using trypan blue staining. On day 5 following transfection, cells were harvested by centrifugation (25 min, 4000 RCF), and cells and supernatants were kept at 4 °C until purification. Enzyme expression was confirmed by SDS-PAGE and Western blot using anti-His antibody to reveal a protein of 88.8 kDa [49].

### 3.4. Purification of B4GALNT1 Using Ni-NTA Column Chromatography

Cells were disrupted by hand homogenization in 50 mM sucrose, followed by centrifugation. His_8_-tagged B4GALNT1 produced by transient transfection in Expi293 cells was purified from the supernatant using a column of 5 mL HisPur Ni-NTA agarose resin slurry (ThermoFisher Scientific, Waltham, MA, USA). The medium was adjusted with 10× media adjustment buffer (200 mM imidazole, 2 M NaCl, and 300 mM sodium phosphate, pH 7.2) to make the final media. The column was equilibrated with 10 column volumes of Buffer A (20 mM HEPES, 300 mM NaCl, 20 mM imidazole, pH 7.2). The sample was adjusted with media adjustment buffer (200 mM imidazole, 2 M NaCl, and 300 mM sodium phosphate, pH 7.2) and was loaded on the equilibrated column, and the flowthrough was collected. The column was washed first with 50 mL of Wash buffer 1 (2 mM HEPES, 30 mM NaCl, 20 mM imidazole, pH 7.2), followed by Wash buffer 2 (2 mM HEPES, 30 mM NaCl, 50 mM imidazole, pH 7.2) and Wash buffer 3 (2 mM HEPES, 30 mM NaCl, 100 mM imidazole). Protein was eluted with 50 mL of Elution buffer (2 mM HEPES, 30 mM NaCl, 300 mM imidazole, pH 7.2). Lastly, the column was washed with 15 mL 2 mM HEPES, 30 mM NaCl, 500 mM imidazole. The eluted protein was concentrated with a 10 kDa MWCO filter (4000× *g*) at 6000 RPM for 10 min at 4 °C (for 10–12 cycles) and buffer-exchanged into 20 mM Tris HCl, pH 7.5 (3 to 4 cycles). The protein concentration was determined using the Bicinchoninic acid assay. Purified protein was stored in aliquots with 20% glycerol at 4 °C, −20 °C, and for long-term storage at −80 °C.

### 3.5. Glycosyltransferase Standard Assays for Human B4GALNT1

B4GALNT1 activity was measured by the transfer of radiolabeled GalNAc from the UDP-[^3^H]GalNAc donor using GM3 as an acceptor substrate. GM3 was dissolved in chloroform/methanol (2:1), which was removed before the assay with a stream of nitrogen. Standard assays contained in a total volume of 50 µL: 20 µL (8 µg protein) purified B4GALNT1 in 20 mM Tris pH 7, 20% glycerol, 0.39 mM UDP-[^3^H]GalNAc (1900 CPM/nmol), 0.2 mM dried acceptor GM3, 40 mM Na-cacodylate buffer pH 7, 0.06% Triton X-100, and 8 mM MnCl_2_. Assay mixtures were incubated at 37 °C for 1 h, and reactions were quenched by freezing. Assays were carried out in duplicate determinations. The negative control assays did not contain the acceptor substrate. The reaction product was isolated via C18 Sep-Pak cartridges and was eluted with MeOH. Radioactivity was measured by scintillation counting.

### 3.6. Bioinformatics

Predicted B4GALNT1 3D structure was obtained from the AlphaFold database, which was used to understand structural features. A docked model of B4GALNT1 binding UDP-GalNAc donor substrate was obtained with MolSoft ICM 3.9. BLAST search was run in Uniprot against the UniRef50 database, with the IDs of the following families mapped against the UniprotKB database: UniRef50_A0A8K1LFD9, UniRef50_C0H8Y3, UniRef50_A0A8I3NPC1, UniRef50_H3DAS0, UniRef50_Q00973, UniRef50_A0AD0GQI1, and UniRef50_A0A974BWB6. Removal of outliers resulted in a final set of 352 sequences, which were aligned using MUSCLE. A consensus sequence, following gap excision, was generated with Weblogo.

### 3.7. Inhibition of B4GALNT1

Inhibition was measured under standard assay conditions for purified B4GALNT1. Compounds were dissolved in MeOH and dried before the addition of the enzyme and other assay components. Positive control assay mixtures contained 0.2 mM GM3 and lacked inhibitor. To determine the IC_50_, compound **QT163** was used at 0.2, 0.4, 0.6, 0.8, and 1 mM concentrations in the assay.

## 4. Conclusions

The aim of this study was to characterize a soluble form of human B4GALNT1, the enzyme that transfers a GalNAc residue in β1-4 linkage to the Galβ1-4 residue of gangliosides GM3, GD3, and other GSLs. The inhibition of this enzyme would reduce the synthesis of GM2, which could be of therapeutic value for patients who accumulate GM2 due to the inability to hydrolyze the GalNAc residue. This inhibition would shift the balance of GSL and may impact cellular functions that rely on specific GSLs on membrane and lipid rafts. Thus, an application of these inhibitors in SRT has to be carefully studied and monitored.

Previous substrate specificity assays [4,33] revealed that ganglioside GM3 containing Neu5Ac was a major acceptor substrate for B4GALNT1 in microsomal membranes and for a soluble recombinant protein. GM3 having Neu5Gc was about 12.8% less active. Our specificity assays using highly purified soluble B4GALNT1 similarly showed that GM3 was the preferred acceptor and confirmed that GD3 exhibited half of that activity, while Lac-Cer and GM1 showed minimal activity. This indicates that the Siaα2-3Galβ1-4Glc- linkage in the acceptor is important for enzyme recognition and that one terminal Sia residue is preferred.

It is not clear yet if milk oligosaccharides having the Siaα2-3Galβ1-4Glc linkage and glycoproteins with Siaα2-3Galβ1-4GlcNAc linkage form acceptors for B4GALNT1 or if the enzyme requires the hydrophobic Glc-Cer moiety in the acceptor. Positively charged residues in the acceptor binding site (R505 and H483) may aid in recognition of Sia, and mutations at these residues may reveal a change in acceptor specificity. Y501 and other hydrophobic amino acids could aid in binding the Cer group of the acceptor.

A comparison of published properties of B4GALNT1 with our work shows that the GM2 synthase activity is dependent on many factors, including the protein sequence and glycosylation, presence of membranes, and assay conditions. These various results allow only an approximate estimate of the activity in vivo, which may differ according to the cell type expressing B4GALNT1.

The aim of this study was to identify inhibitors for B4GALNT1 that may be developed for treatment of GM2 gangliosidosis but not cause symptoms of SPG. The compounds we chose might be appropriate for this purpose since they inhibited B4GALNT1 activity between 40 and 94%. A complex of B4GALNT1 docked with its donor substrate UDP-GalNAc showed amino acids near the docking site that may play a role in UDP-GalNAc binding and catalysis. This information, together with protein structure analysis of B4GALNT1 to confirm the role of conserved amino acids, will aid in the design of additional, specific, non-covalent inhibitors suitable for SRT. Studies of drug development should also include the kinetics of inhibition, test dosage, and toxicity, and address drug delivery to neurons that accumulate GM2.

## Figures and Tables

**Table 1:** Amino acid residues important for activity of human B4GALNT1.

MSA	Variants	Activity of Mutants	References
K284N	SPG		[25]
R288	SPG		[24]
R300C	SPG		[24]
D313A	SPG		[24,39]
V352		167%	[38]
W354		24%	[38]
D356		nd	[38]
D357			
D358		nd	[38]
(F392)			
C429			
D433A	SPG		[24]
N437K	SPG	nd	[39]
F438L	SPG		[39]
F439	SPG		[24]
A441E	SPG	nd	[39]
P453H	SPG	nd	[39]
C476			
H483			
K486			
Y501			
R505H	SPG	nd	[25]
Q514			
A516	SPG		[24]
R519P/W	SPG	nd	[26,39]

**MSA**, multiple sequence alignments, showing conserved amino acids determined by BLAST. Numbers in brackets denote less conserved residues. SPG mutants are included. **Variants**, natural variants in patients with hereditary spastic paraplegia (SPG). Mutations in Variants affect highly conserved amino acids, with the exception of W354, A516, and R519, which are not highly conserved in human B4GALNT1. **Activity of mutants**, created by single amino acid replacements. nd, not detected.

**Table 2:** List of bis-imidazolium inhibitors tested for activity of purified B4GALNT1.

Inhibitor	n	m	% Inhibition
**QT149**	22	1	90
**QT163**	6	11	80
**QT160**	10	10	89
**QT161**	10	11	84
**QT162**	10	12	94
**QT166**	16	7	92
**QT169**	16	10	74
**QT170**	16	11	40
**QT171**	16	12	47
**612**	-	-	44

## Data Availability

The authors will make data available upon request.

## Supplementary Materials

The following supporting information can be downloaded at: https://www.mdpi.com/article/10.3390/molecules30173615/s1, Figure S1: Expression of His8-tagged B4GALNT1 in Expi293 cells.; Figure S2: SDS-PAGE after purification of His8-tagged B4GALNT1 expressed in Expi293 cells and secreted into the culture medium.; Figure S3. Detergent effect.
