# Supplementary material for: Characterization of Human Recombinant β1,4-GalNAc-Transferase B4GALNT1 and Inhibition by Selected Compounds
Source: Molecules. 2025 Sep 4;30(17):3615. doi: 10.3390/molecules30173615 (PMC12430364; doi:10.3390/molecules30173615)
Supplement: Supplementary file 1 [file molecules-30-03615-s001.zip › molecules-3763095-supplementary.pdf]

Supplementary Figures:

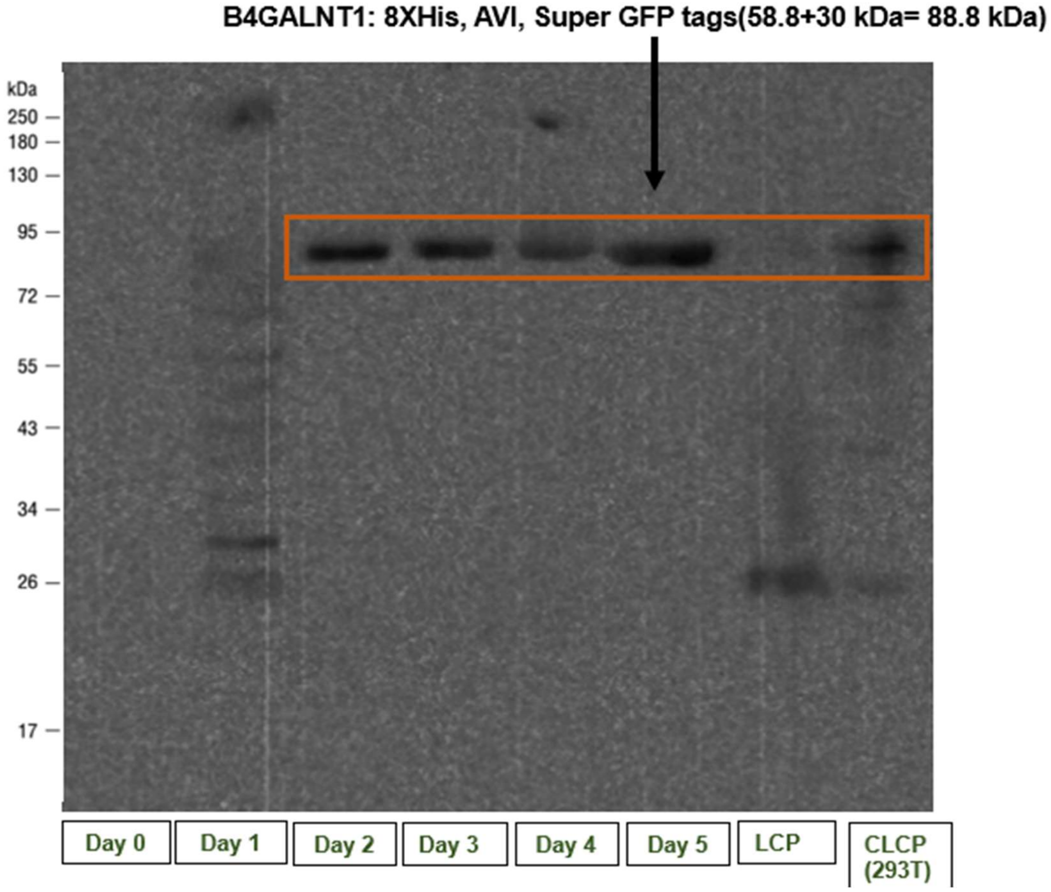

Figure S1: Expression of His8-tagged B4GALNT1 in Expi293 cells.

Western blot using anti-His antibody confirmed expression of His8-tagged B4GALNT1 in Expi293 suspension cells. Soluble protein was identified in the medium at theoretical size at 88.8 kDa. Samples were collected at Day 0, Day 1, Day 2, Day 3, Day 4 and Day 5 after expression (Medium), LCP: Lysed cell pellet has no visible band, CLCP (Control Lysed cell pellet from B4GALNT1-expressing HEK293T cells) shows a faint band in the pellet. This shows that B4GALNT1 is soluble when expressed in Expi293 but is in the insoluble fraction when expressed in HEK293T cells.

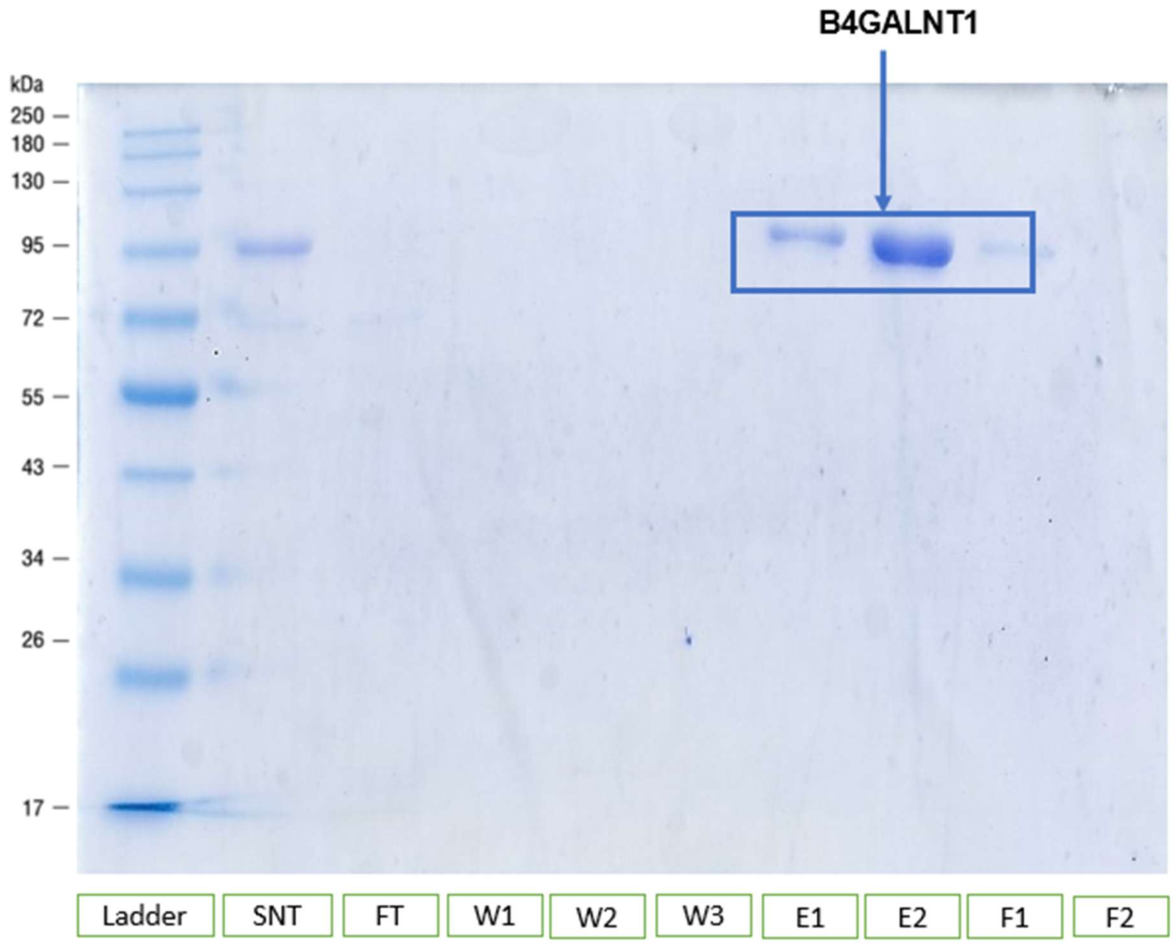

Figure S2: SDS-PAGE after purification of His8-tagged B4GALNT1 expressed in Expi293 cells and secreted into the culture medium. B4GALNT1 appeared on a 12% SDS-PAGE gel in E1, E2 and F1 fractions with the strongest band in the E2 fraction, corresponding to the theoretical size of 88.8kDa. SNT, supernatant; FT, flow-through; W1, W2, W3, wash fractions. Protein was eluted with 300 mM (E1) and 500 mM imidazole (E2, F1, F2).

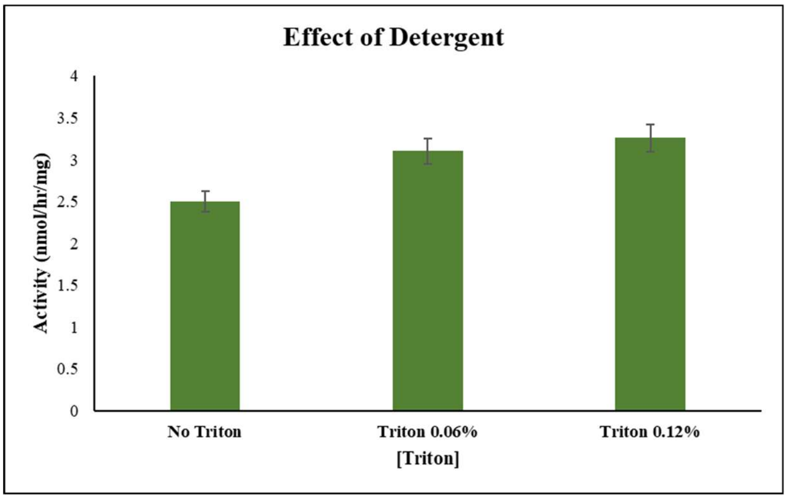

Figure S3. Detergent effect. The bar graph shows the effect of Triton from 0 to 0.12 % concentrations on the activity of purified B4GALNT1 with GM3 as acceptor substate. Error bars indicate variations between duplicate determinations.
